# Impact of Instrumentation Technique on Endodontic Treatment Quality in Curved Molars: A Retrospective Analysis

**DOI:** 10.1111/aej.12952

**Published:** 2025-05-26

**Authors:** Carolina Clasen Vieira, Luciéli Andréia Zajkowski, Daniele Assumpção Prado, Fábio de Almeida Gomes, Patrícia Maria Poli Kopper, Katerine Jahnecke Pilownic, Daiana Elisabeth Böttcher, Tatiana Pereira Cenci, Erick Miranda Souza, Fernanda Geraldo Pappen

**Affiliations:** ^1^ Graduate Program in Dentistry Federal University of Pelotas Pelotas RS Brazil; ^2^ Graduate Program in Dentistry Federal University of Rio Grande Do Sul Porto Alegre RS Brazil; ^3^ School of Dentistry Universidade de Fortaleza, UNIFOR Fortaleza CE Brazil; ^4^ Graduate Program in Dentistry School of Health and Life Sciences, Pontifical Catholic University of Rio Grande Do Sul Porto Alegre Brazil; ^5^ School of Dentistry Federal University of Maranhão, UFMA São Luis MA Brazil

**Keywords:** dental students, instrumentation, root canal preparation, technical quality, treatment failure

## Abstract

This cross‐sectional study evaluated technical failures in root canal treatments (RCTs) of curved molars (> 10°) performed by undergraduate students and their association with instrumentation techniques. Evaluations included filling extension, density, taper and ledge/zip formation. Bivariate analysis (chi‐square, 5% significance) calculated odds ratios (OR) and 95% confidence intervals (CI). Among 188 RCTs, manual instrumentation had higher risks of ledge/zip formation, inadequate density, taper and underfilling, while reciprocating techniques showed more overfilling. Root curvature did not influence failure rates. The study showed 100% power for detecting underfilling, inadequate density and taper; 97.98% for overfilling and 71.11% for ledge/zip formation. Instrumentation technique significantly impacted failure frequency, with manual techniques associated with more complications.

## 1 Introduction

One of the primary factors contributing to endodontic treatment failure is the complexity of root canal system anatomy. The intricate structure of root canals often hinders complete canal cleaning using current technologies, potentially leaving viable microorganisms behind and leading to periapical disease. Unsurprisingly, the incidence of technically unsatisfactory treatments and endodontic failures is higher in molars than in other dental groups due to the complex anatomical configuration of their root canal systems, including variable angles and radii of root curvatures. These anatomical challenges significantly affect the adequacy of cleaning, shaping and filling [1].

The technical challenges posed by complex root canal anatomies are often heightened by the operator's skill level. Studies assessing factors such as root canal filling length, density, taper and the occurrence of procedural accidents, including ledge or zip formation, root perforation and instrument fractures, report that lower success rates are prevalent in multi‐radicular teeth treated by undergraduate students [2, 3, 4].

To improve technical outcomes and shorten the learning curve for students, dental schools worldwide are increasingly incorporating advanced technologies into their curricula. These include apex locators, rotary and reciprocating nickel–titanium instruments and single gutta‐percha cones with greater taper [5]. Reciprocating instruments have shown promise for use by less experienced practitioners. They simplify the learning curve for root canal instrumentation and significantly reduce the incidence of anatomy‐related accidents, especially in curved canals [6, 7].

The quality and efficiency of root canal filling achieved by undergraduate students using mechanised systems are superior to those achieved with manual instrumentation [8]. Despite the promising results of incorporating reciprocating instrumentation into preclinical and clinical training, manual instrumentation using stainless steel files remains the most common technique taught in Brazilian universities. However, the poor outcomes often observed in treatments performed by undergraduate students may be attributed to the high operator sensitivity required and the steep learning curve associated with manual techniques.

This study, therefore, aimed to evaluate the frequency of technical failures in the root canal treatment of curved molars performed by undergraduate students and their association with different instrumentation techniques: manual versus reciprocating. The null hypothesis was that there would be no association between the frequency of technical failures in molar root canal treatment and the instrumentation techniques employed.

## 2 Materials and Methods

### 2.1 Ethical Considerations and Study Design

This study received approval from the Local Research and Ethics Committee (CAAE #06198819.6.2001.5317). The confidentiality of the patient's personal information was strictly maintained to protect their identities.

This study is a cross‐sectional observational evaluation examining the frequency of technical failures in root canal treatments (RCTs) of curved molars performed by undergraduate students. Findings are reported following the STROBE guidelines [9]. Additionally, the study investigates the association between these failures with instrumentation techniques (manual vs. reciprocating).

### 2.2 Cases Selection

This study involved a primary randomised controlled trial focusing on molar teeth, conducted by fifth‐year undergraduate students. Endodontic professors supervised all RCTs. Periapical radiographs were obtained, and the images were digitised using an HP ScanJet G4050 scanner (HP Brasil, Barueri, SP, Brazil), and stored as TIFF files at a resolution of 300 DPI. Case selection followed a census approach. The evaluation was conducted in the mesial roots of the upper and lower molars. Inclusion criteria required complete clinical records and properly achieved and processed final periapical radiographs, to minimise length and angle distortions. The periapical radiographic images should show the entire dental crown and roots and approximately 2–3 mm of the periapical region.

**FIGURE 1:**
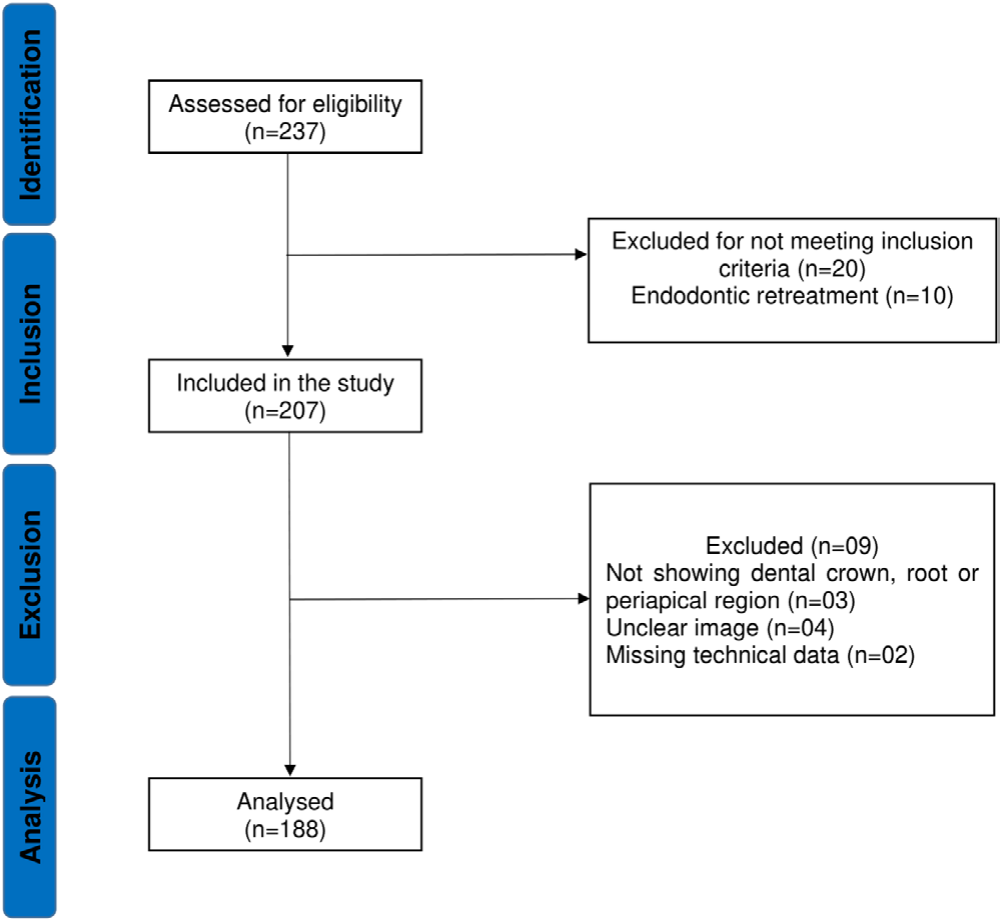
Flowchart of the study sample reported according to the STROBE guideline.

Exclusion criteria included unclear dental crown or root images, inability to determine the occlusal plane, teeth with immature root development, non‐molar teeth and endodontic re‐treatments. Additionally, records with missing data on technical variables, such as instrumentation and filling techniques, were excluded (Figure 1).

The statistical power calculation was determined using OpenEpi version 3.01 (Andrew G. Dean & Kevin M. Sullivan, Atlanta, GA, USA).

### 2.3 Evaluation of the Degree of Root Curvature

The degree of root curvature in the mesial roots of the included molars was accessed. A single trained and calibrated examiner analysed the final radiographic images, focusing on the degree of root curvature and the technical quality of the root canal filling. Before evaluation, a consensus regarding all variables was established among the senior researchers and the examiner. To assess intra‐examiner agreement, 10% of the cases were randomly selected and re‐analysed 2 weeks later.

The evaluation occurred in a dark room on a 19‐in. LCD monitor (64‐bit, colour, 1600 × 1220 pixels) with ImageJ software (version 1.52a, National Institutes of Health, Bethesda, MD, USA). The examiner had access to various image‐editing tools, including density, contrast, gamma curvature and magnification, without a time limit for analysing each image.

The degree of root curvature was measured by drawing two lines on the X‐ray images: the first line was parallel to the long axis of the canal and the second line extended from the apical foramen to the point where the root curvature began. The acute angle formed by these lines was measured according to Schneider [10]. The lines were traced using Adobe Photoshop (version 7.0) and root curvature was quantified using ImageJ software. Only roots exhibiting moderate or high difficulty levels, defined as having curvatures greater than 10° [11], were included in the data analysis.

### 2.4 Technical Procedures

All endodontic and restorative procedures were performed under controlled and standardised conditions, under the supervision of a professor specialist in endodontics. The students received expository theoretical classes and laboratory training in both manual and reciprocating techniques and received instructions on correctly filling out the patient's clinical records.

In a part of the sample, the chemical–mechanical preparation was performed with hand files using the crown‐down technique with cervical preflaring using Gates–Glidden drills (Dentsply Maillefer, Ballaigues, Switzerland), while in other part of the sample, RCT was performed using the reciprocating instrumentation technique, with Reciproc files (VDW, Munich, Germany).

### 2.5 Data Collection

A single researcher performed data collection. The variables extracted from the patients' clinical records included gender (female or male), tooth position (upper or lower molar), initial diagnosis (vital pulp or necrotic pulp), root canal preparation technique (reciprocating files or hand files) and obturation technique (single cone or cold lateral condensation).

From the radiographic analysis, the following variables were assessed: root canal filling length (adequate, when the filling was between 0 and 2 mm from the apex; underfilled, when the filling was ≥ 2 mm from the apex and overfilled, when root canal filling length was beyond the apex), density (adequate or inadequate), taper (adequate or inadequate) and the occurrence of ledges or zip (absent or present).

### 2.6 Evaluation of the Factors Associated With Technical Failures

The root canal filling quality assessment was performed based on the parameters described in the ESE's quality guideline for RCT [12]. The evaluation considered the root canal filling length, density and taper, as well as the occurrence of accidents (Table 1).

**TABLE 1:** Parameters for evaluating the technical quality of root fillings.

Variable	Standard	Definition
Root canal filling length	Adequate	Endodontic material placed 0–2 mm from the apex
	Underfilled	Endodontic material more than 2 mm from the apex
	Overfilled	Endodontic sealer or gutta‐percha beyond the apex
Root canal filling density	Adequate	Uniform density, without gaps in the filler
	Inadequate	Non‐uniform density, with clear presence of gaps in the filler
Root canal filling taper	Adequate	Uniform taper from the coronal third to the apical portion, respecting the anatomy of the root canal
	Inadequate	Excessive, insufficient or irregular taper
Occurrence of accidents	Absent	No accidents or intercurrences; maintenance of the root canal's original path
	Ledge/zip formation	Occurrence of ledges and zips from the original path of the root canal

To evaluate the extent of the obturation, the radiographs were re‐sized with a magnification of 10 times the original size, and the distance between the obturation and the radiographic apex was measured. The evaluation was performed using the ImageJ software (version 1.52a, National Institutes of Health, Bethesda, MD, USA).

### 2.7 Statistical Analysis

Descriptive analysis (frequencies, mean, minimum and maximum values and standard deviation) was performed for ledge/zip formation, inadequate density, inadequate taper, underfilled and overfilled root canals as well as for tooth position and instrumentation technique. Bivariate associations between the dependent variables with tooth type, root canal and instrumentation technique were analysed using the chi‐square test. The level of significance was set at 5%. Odds ratios (OR) and 95% confidence intervals (CI) were calculated. Data were analysed using STATA 14 (Stata Corporation, College Station, TX, USA).

## 3 Results

The intra‐observer agreement for qualitative parameters was assessed using Cohen's kappa coefficient, which resulted in values equal to or greater than 0.88. An intra‐observer agreement was determined using the intra‐class correlation coefficient for root curvature measurements, yielding an index of 0.84.

A total of 188 root canal treatments (RCTs) from 179 patients (93 females, 86 males) were analysed, with a mean age of 38.85 years (±19.42). The baseline characteristics of the sample are detailed in Table 2. Most treated teeth were lower molars (71.4%) and presented with pulp necrosis (66.5%). Conventional manual instrumentation using hand files was employed in 102 (49.5%) cases, while 104 (50.5%) cases were instrumented with a reciprocating technique. Regarding root canal curvature, 131 teeth (63.6%) exhibited a curvature degree between 10° and 30°, while 57 teeth (27.7%) had curvatures exceeding 30°.

**TABLE 2:** Baseline characteristics of patients within the groups.

Factors	
Age (mean, SD)	38.85 ± 19.42
Gender, *n* (%)	
Female	93 (52.0)
Male	86 (48.0)
Tooth type, *n* (%)	
Lower molar	134 (71.3)
3 canals	111 (82.8)
4 canals	23 (17.2)
Upper molar	54 (28.7)
3 canals	44 (81.5)
4 canals	10 (18.5)
Number of canals	
Initial diagnosis, *n* (%)	
Vital pulp	64 (34.0)
Necrotic pulp	124 (66.0)
Root canal preparation, *n* (%)	
Reciprocating	96 (51.1)
Manual	92 (48.9)
Obturation technique, *n* (%)	
Single cone	92 (48.9)
Lateral condensation	96 (51.1)

**FIGURE 2:**
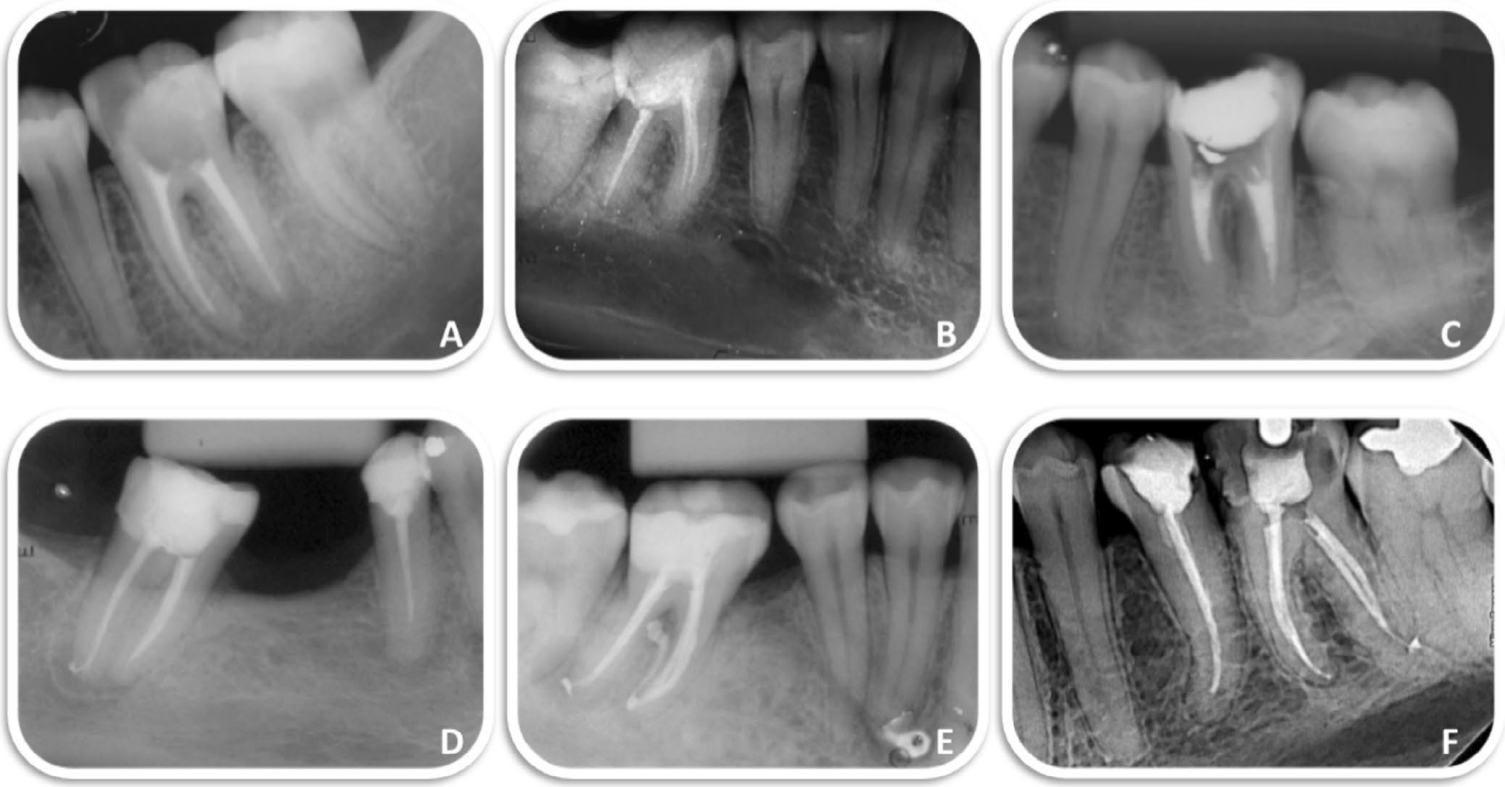
Post‐operative radiographs demonstrating treated canals using (A–C) manual technique and (D–F) reciprocating technique. The reciprocating technique exhibits superior maintenance of working length and root canal curvature, with greater respect for anatomical structure and reduced dentine loss. Obturation appears more homogeneous, though with occasional overfilling. In contrast, the manual technique resulted in loss of the working length and deviations from root canal curvature.

The most frequently observed technical failures in root canal obturation were inadequate density and taper, followed by short filling lengths (Figure 2). The frequencies of these failure parameters concerning pre‐ and intra‐operative variables are summarised in Table 3. Bivariate analysis revealed that the instrumentation technique was significantly associated with all evaluated outcome criteria for root canal filling technical failures (*p* < 0.05).

**TABLE 3:** Factors associated with the technical failures of root canal filling in molars.

	Underfilled	*p* ^a^	Overfilled	*p* ^a^	Inadequate density	*p* ^a^	Inadequate taper	*p* ^a^	Ledge/zip occurrence	*p* ^a^
Tooth										
Lower molar	40 (29.9)	0.976	18 (13.4)	0.050	44 (32.8)	0.948	44 (32.8)	0.948	22 (16.4)	0.205
Upper molar	16 (29.6)		2 (3.7)		18 (33.3)		18 (33.3)		5 (9.3)	
Curvature^b^										
Moderate	38 (29.0)	0.723	12 (9.2)	0.319	45 (34.4)	0.544	43 (32.8)	0.946	17 (13.0)	0.412
Severe	18 (31.6)		8 (14.0)		17 (29.8)		19 (33.3)		10 (17.5)	
Instrumentation										
Reciprocating	6 (6.3)	< 0.001	18 (18.8)	< 0.001	2 (2.1)	< 0.001	6 (6.3)	< 0.001	8 (8.3)	0.016
Manual	50 (54.3)		2 (2.2)		60 (65.2)		56 (60.9)		19 (20.7)	
Obturation										
Single‐cone	6 (6.5)	< 0.001	16 (17.4)	0.003	2 (2.2)	< 0.001	6 (6.5)	< 0.001	8 (8.7)	0.015
Lateral condensation	50 (52.1)		4 (4.2)		60 (62.5)		56 (58.3)		19 (19.8)	

^a^ Chi‐square test.

^b^ Moderate curvature: curvatures between 10° and 30°; severe curvature: curvatures exceeding 30°.

The study power was calculated for all dependent variables, including ledge/zip formation, underfilling, overfilling, inadequate density and inadequate taper. Calculations were based on the following parameters: a 95% confidence interval (CI); exposure to root canal instrumentation with reciprocating files (*n* = 106) and non‐exposure, involving instrumentation with hand files (*n* = 102). The study power was determined to be 100% for underfilling, inadequate density and inadequate taper; 97.98% for overfilling and 71.11% for ledge/zip formation.

## 4 Discussion

This study analysed data from endodontic clinical and radiographic records of teeth treated by undergraduate students. The findings are essential for assessing the learning outcomes and technical quality of treatments performed during undergraduate education and identifying areas requiring improvement in the teaching of endodontics. The null hypothesis was rejected, as the instrumentation technique showed a significant association with factors contributing to technical failures.

According to the European Society of Endodontology [12], root canal instrumentation should yield a tapered and uniform shape from the cervical third to the apex. The assessment of RCT quality should include adequate working length—defined as the closest possible to the apical constriction—complete filling of the root canal with a (semi‐)solid material and an endodontic sealer, absence of space between the filling material and root canal walls (indicating adequate density) and tapered root canal shape from crown to apex (indicating adequate taper). Nevertheless, in this study, the parameters for evaluating the frequency of failures in endodontic treatments were based on this recommendation.

There are several methods to determine the degree of root curvature, which can be assessed through periapical radiographs, despite their limitations in presenting only two dimensions, or cone‐beam computer tomography (CBCT) [13]. In this study, the method used for curvature measurement was based on the classical technique described by Schneider [10] and classified according to the American Association of Endodontics (AAE) [11].

The AAE classification divides canal and tooth root morphology into three categories based on the degree of difficulty for treatment: minimal or mild (curvatures less than 10°), moderate (curvatures between 10° and 30°) and high or severe (curvatures greater than 30°), and curvatures less than 10° are considered to have minimal difficulty, making treatment outcomes more predictable and achievable by competent professionals, even those with limited experience, such as undergraduate students. Curvatures greater than 10° may present moderate to high difficulty, establishing challenges even for experienced professionals and specialists. Therefore, such cases may not be encouraged for undergraduate students [11].

Even though endodontic procedural errors are not the direct cause of treatment failure, when such errors occur, the technical quality rates of endodontic treatment are diminished [14]. Procedural errors can compromise root canal cleaning and shaping, leading to incomplete obturation, which may affect the treatment's overall success. Accidents during instrumentation are more common in molars and curved root canals [1]. Furthermore, root curvature is identified as a determining factor contributing to deviations [14, 15], which occurred in 13.6% of the molars in our study. This is attributed to the higher prevalence of narrow and curved root canals in molar teeth, making them more challenging for students.

The molars analysed in this study exhibited root curvatures exceeding 10°, categorised as moderate to high difficulty by the AAE [11]. Achieving adequate canal volume enlargement with conical, centred preparation poses significant challenges in curved canals [16]. These challenges are influenced by the curvature angle and factors such as instrumentation kinematics, instrument flexibility and diameter and the selected technique [17]. In this context, reciprocating instrumentation demonstrates notable advantages. The simplicity of this method, combined with the use of Ni‐Ti instruments, enhances safety and efficiency, minimising procedural errors. In contrast to previous studies comparing canals with minimal (< 10°) versus moderate to severe (> 10°) curvatures [18, 19], our findings demonstrate that root canal angulation did not significantly influence outcomes when analysing exclusively moderate to severe curvatures (> 10°). Consequently, when considering the results collectively, root angulation did not appear to exert a significant influence.

Our results revealed a strong association between the instrumentation technique and all variables analysed to evaluate technical failures. Among these, inadequate density was the most frequently observed failure, followed by inadequate taper and insufficient root canal obturation, aligning with previous findings in the literature [4, 20]. Additionally, the adjusted analysis indicated that manual kinematics were more likely to result in underfilling, inadequate density and taper and an increased risk of ledge formation. Despite these limitations, using stainless steel hand files in the crown‐down technique remains widely practised and taught in undergraduate courses [2].

Previous studies comparing automated and manual techniques have consistently reported a higher incidence of procedural errors with manual instrumentation, including ledge or zip formation, canal transportation and instrument fracture, alongside lower success rates [4, 21], particularly among less experienced operators [22]. The extensive number of steps involved in hand file techniques, combined with the considerable stiffness of stainless steel files, may contribute to these outcomes [23].

Conversely, numerous advantages of Ni‐Ti rotary instruments over stainless steel hand files have been demonstrated, including better preservation of the original canal shape and curvature, fewer catastrophic errors and reduced treatment time [21]. Although the evidence in the literature regarding the impact of automated instrumentation on endodontic treatment quality remains limited, particularly for reciprocating files, these instruments are viable alternatives for undergraduate students. Their shorter learning curve and lower incidence of accidents and complications make them especially suitable for beginners [7, 22].

Mechanised Ni‐Ti instruments have been shown to shape root canals more effectively than hand files [21]. However, due to their ease of handling and, more importantly, the lack of attention to reference points—particularly among undergraduate students—over instrumentation is frequently reported with reciprocating instrumentation techniques [23]. Consequently, apical foramen widening and extrusion of root canal filling material are more common with reciprocating techniques, as demonstrated in our study.

Even though persistent discussion about whether sealer extrusion or unintentional overfilling could trigger chronic inflammatory responses and reduce endodontic success rates, recent data have concluded that factors such as the type of extruded material, its resorption or persistence are not directly associated with unfavourable outcomes [24].

In addition to root canal filling length, another crucial aspect to evaluate in RCT is the density of the root canal obturation. Optimal density is achieved when a homogeneous, radiopaque image is observed radiographically in the root canal filling. The literature has reported that less dense and non‐homogeneous obturations are associated with a higher risk of infiltration, thereby increasing the likelihood of unfavourable RCT outcomes, though homogeneous filling without voids is strongly correlated with improved clinical success [25].

Inadequate density often results from deficiencies in chemical–mechanical preparation and, more importantly, improper filling techniques. In our study, all cases of manual instrumentation were filled using the cold lateral condensation technique, which explains why 63.7% of these cases were classified as having inadequate density. In contrast, above 95% of cases treated with the reciprocation technique were filled using the single‐cone technique and only 1.9% presented unsatisfactory density. The formation of voids, caused by placing the digital spacer to place accessory cones, is a common issue when using the cold lateral condensation technique [2].

This study has certain limitations. The first relates to the radiographic interpretation of endodontic treatments based on periapical radiographs. As is well established, radiographs provide a two‐dimensional representation of a three‐dimensional structure, which can hinder the differentiation of overlapping anatomical features. Additionally, root lengths and obturations may not be accurately represented due to distortions in the vertical angulation of radiographic incidence. Proper processing and storage of radiographs are essential to ensure high‐quality images for reliable analysis. The second limitation arises from the retrospective nature of this study, which prevents control over certain variables. Factors such as the individual skill levels of dental students, patient behaviour, differences in faculty assistance and case difficulty may have varied and remain uncontrolled.

The literature indicates that RCT success rates improve with the operator's clinical experience, with significantly higher success observed when using automated Ni‐Ti instruments [26]. To enhance the clinical performance of undergraduate dental students and improve the quality of treatments delivered to patients, revisions to the endodontics curriculum are necessary. Curricular changes should be implemented even in preclinical courses, integrating new technologies, such as foraminal locators and Ni‐Ti instruments with reciprocating kinematics [27]. These improvements can enable safer, faster and higher quality RCTs, reducing the likelihood of accidents and complications.

Moreover, dental schools should develop teaching and clinical practice strategies tailored to the complexity of instrumentation and obturation in each case, as well as the anatomical challenges posed by individual dental elements.

## 5 Conclusions

The instrumentation technique significantly influenced the frequency of technical failures in molar root canal treatments performed by undergraduate students, with the reciprocating technique demonstrating superior clinical outcomes compared to the manual technique.

## Author Contributions

**Carolina Clasen Vieira:** methodology, investigation, writing – original draft. **Luciéli Andréia Zajkowski, Daniele Assumpção Prado:** methodology, investigation, data curation. **Fábio de Almeida Gomes, Erick Miranda Souza:** conceptualisation, methodology, writing – review. **Patrícia Maria Poli Kopper:** conceptualisation, writing – review, supervision. **Katerine Jahnecke Pilownic:** conceptualisation, writing – original draft, review. **Daiana Elisabeth Böttcher:** data curation, writing – review. **Tatiana Pereira Cenci:** supervision, writing – review. **Fernanda Geraldo Pappen:** conceptualisation, methodology, formal analysis, data curation, writing – review, supervision, resources, project administration.

## Disclosure

Authorship declaration: All authors have approved the final version and consent to its submission. All authors have contributed significantly and agree with the content of the manuscript.

## Conflicts of Interest

The authors declare no conflicts of interest.

## Data Availability

The data that support the findings of this study are available from the corresponding author upon reasonable request.
